# The impact of vulnerability and exposure to pervasive interprofessional incivility among medical staff on wellbeing

**DOI:** 10.3389/fpubh.2023.1168978

**Published:** 2023-07-14

**Authors:** Antoinette Pavithra, Russell Mannion, Ling Li, Johanna Westbrook

**Affiliations:** ^1^Faculty of Medicine, Health and Human Sciences, Centre for Health Systems and Safety Research, Australian Institute of Health Innovation, Macquarie University, Sydney, NSW, Australia; ^2^Health Services Management Centre, School of Social Policy, College of Social Sciences, University of Birmingham, Birmingham, United Kingdom

**Keywords:** healthcare workforce, incivility, organizational culture, medical staff, self-organizing social system, human interaction impact modeling, interprofessional behavior, staff wellbeing

## Abstract

**Introduction:**

Traditional methods for modelling human interactions within organisational contexts are often hindered by the complexity inherent within these systems. Building on new approaches to information modelling in the social sciences and drawing on the work of scholars in transdisciplinary fields, we proposed that a reliable model of human interaction as well as its emergent properties can be demonstrated using theories related to emergent information.

**Methods:**

We demonstrated these dynamics through a test case related to data from a prevalence survey of incivility among medical staff. For each survey respondent we defined their vulnerability profile based upon a combination of their biographical characteristics, such as age, gender, and length of employment within a hospital and the hospital type (private or public). We modelled the interactions between the composite vulnerability profile of staff against their reports of their exposure to incivility and the consequent negative impact on their wellbeing.

**Results:**

We found that vulnerability profile appeared to be proportionally related to the extent to which they were exposed to rudeness in the workplace and to a negative impact on subjective wellbeing.

**Discussion:**

This model can potentially be used to tailor resources to improve the wellbeing of hospital medical staff at increased risk of facing incivility, bullying and harassment at their workplaces.

## 1. Introduction

Over the last decade, researchers have extended the principles of information theory and quantum mechanical formalism to the social sciences (1–3). These innovative lines of enquiry into the nature of human systems have allowed social scientists to explore theoretical frameworks that can help explain the supposed inscrutability inherent within complex human assemblages (4, 5). The study of disruptive or uncivil human behavior within organizations is an area where such theoretical developments may shed light and aid the development of sustainable solutions. Characterizing an organization as an infological system allows for the study of the material, symbolic and system of structures that comprise it (3, 6). For instance, interprofessional behaviors that are enacted, perceived, and received as professional or unprofessional can be viewed as a combination of moralized, values-laden, and socio-culturally constituted interactions. Consequently, unprofessional behaviors in healthcare organizations are not only emergent properties within human systems but are also profoundly influenced by context. For example, when a senior medical specialist is rude to their intern and publicly corrects them for not responding decisively in a clinical situation, the senior medical specialist may view their own behavior as warranted. However, the intern’s experience of the same event may result in psychological harm because of feeling humiliated and belittled, particularly if such interactions occur repeatedly. Therefore, identifying the complex factors that contribute to negative interprofessional staff behaviors, and mitigating their negative impacts on individuals and within organizations can prove challenging.

Traditional frameworks used to study the emergent properties of human interactions such as unprofessional behaviors demand a reductionism of the phenomenon to derive statistically valid evidence (7). Innovative approaches that aim to provide a unified theory of information appear to offer an alternative approach that circumvents this reductionism and progresses the scholarship in this field beyond the dichotomy of determinism. Large organizations are fundamentally designed and structured on the premise of functionalist accounts, therefore framing emergent phenomenon such as unprofessional staff behavior as aberrant or bad (7, 8). Consequently, addressing aberrant events then demands inordinate amounts of resources and modularity in solutions that may not adequately address the related factors that enable these aberrations to remain. The prevalence of unprofessional behaviors within complex and dynamic organizations such as hospitals has been characterized as an endemic and an entrenched phenomenon typical of large healthcare systems (9). The phenomenon of staff unprofessional behavior in hospitals has been portrayed as difficult to model or predict within literature related to patient safety and healthcare organizational management studies. Traditionally, only statistical, qualitative or a combination of these two methods have been used to understand and describe the prevalence of unprofessional behaviors within organizations, and how these cultural elements emerge, unfold, and further inform the behavior of people within professional systems.

Foundational arguments within mathematical anthropology assert that patterns of behaviors not only express shared ideas, beliefs, values, but also demonstrate the structural organization of these human systems (10). In this sense, professionalization, group identity and organizational cultures within occupational groups may reflect features of kin structures and systems of behavior, class organization (11). Indeed, Lévi-Strauss had argued that within the future of kinship studies, not only would structures be composed of commutative classes and networks, but would also be composed of “unpredictable events, whose statistical distribution…will show regularities and provide meaningful clues” (12). In some studies of professional systems in healthcare, demographic similarities, and socio-cultural characteristics among individuals within networks have been described as endowing the self-organizing properties of kinship systems to these professional and practice networks (13). In this context, the emergence of behavioral patterns such as unprofessional behaviors within medical practitioner communities does appear to fit Strauss’ conceptualization of unpredictable events that occur with regularity in social systems, and provides us clues about how these groups are organized and structured. However, within contemporary scholarly literature related to unprofessional behaviors within organizations, there is an absence of mathematical representations about the structural implications of how culture and behavior inform professional human systems. In an effort to understand whether this gap can be filled, it may be worthwhile to combine descriptive statistical methods of study, and data collected using these methods with mathematical representational efforts to ascertain whether the behavioral artifacts of professional practitioner systems and cultures can be modeled to describe how these systems are organized and maintained.

Within organizational improvement studies, staff negative behaviors have typically been viewed through the lens of a subject-object dialectic where perpetrators, victims and the organization are seen as inter-related but, ultimately, distinct. An alternative and possibly better-suited approach to viewing relational and behavioral dynamics in organizations could be derived by applying the paradigm of self-organizing systems. This approach has been discussed by Hofkirchner in “Emergent Information: A Unified Theory of Information Framework” (7). Within this approach, emergent dynamics within self-organizing systems have been described as an evolutionary system where,

“s_e_ = (defined as) a collection of

(1) elements E that interact such that,

(2) relations R emerge that – because of providing synergistic effects – dominate their interaction in (3) a dynamics D”

Therefore, if subcultures within groups of staff and sub-professional units within hospitals are viewed as artifacts of self-organizing systems, the prevalence of unprofessional behaviors such as incivility and rudeness within a hospital can be defined as a by-product of pre-existing dynamics between the members of a professional socio-cultural system. If conditions that give rise to these emergences could all be observable in theory, the prevalence of these unprofessional behaviors could be predicted and therefore, attenuated or even prevented.

Through this article, we aim to demonstrate the emergence of system dynamics as an artifact of self-organization among medical staff. We argue that the emergence of uncivil behaviors such as rudeness, that arise in interactions between hospital medical professionals, can be modeled through a composite of each staff member’s individual profile that may make them vulnerable to, or protect them from, system dynamics. We posit that every member within the system can be characterized as a combination of demographic traits that impact their experience within the workplace (14). While not every biographical characteristic can be realistically or reliably measured due to limitations presented by traditional research methods, practice and resources, characteristics that can be captured and have been used for this study are, age (a), gender (g), professional sub-role and associated status (s), length of employment (l) within an organization and type of funding (f) that is used to operate the organization, i.e., private or public (which may indicate the sufficiency of other resources). Each staff member’s profile (p) can then be represented as a collection of their a, g, l, and f,


p=[a,g,s,l,f]

Assuming that self-reported scores for the negative impact on wellbeing from an interpersonal or interprofessional event can be used to test our argument, we could reason that the impact (i) as well as exposure to unprofessional behaviors (e) should be proportional to the composite of the profile of each staff member. This dynamic can therefore be represented as,


p∝[e,i]

## 2. Methods

As part of an evaluation of a culture change intervention across seven hospitals in three Australian states, a large- scale baseline survey of incivility was conducted in 2017/18 (15). A total of 5,178 staff responded to the survey seeking to establish the prevalence of 26 unprofessional behaviors. Among all respondents, 546 were identified as medical professionals from sub-roles such as surgical staff specialist, medical staff specialist, visiting medical officer, registrar, career or hospital medical officer, medical fellow, resident, or intern. Secondary analysis was undertaken on the data collected from the survey and has been used to report findings presented in this article. The analysis reported in this article pertains only to these 546 medical professionals. Staff indicated the gender they identified with as male, female, other. They were also provided the option of “prefer not to say.” Respondents were surveyed on temporal factors such as their length of employment at their current hospital, and their age and gender. Responses indicated as “prefer not to say” or missing responses for gender, length of employment and age were excluded from the analysis. If respondents indicated their sub-roles, these were factored into the analysis. Where this information was not available, a mean score from available data was allotted to respondents. Responses related to the extent of exposure to one item enquiring about rudeness, namely, “In the past 12 months, how often have you experienced the following staff behaviors in this hospital – being spoken to rudely” (scored on a seven-point Likert scale that graded responses as, “never, 1–2 times/year, every few months, around monthly, weekly, daily, and multiple times daily”) were extracted and used in this study. Responses to questions related to the other 25 unprofessional behaviors were not included in this analysis as this is intended to be a test case using the event of being spoken to rudely within professional contexts. Responses to the question, “Thinking about your experience of unprofessional staff behaviors in this hospital, to what extent do you believe they have had a negative impact on – you and your wellbeing” were reported on a five-point Likert scale with responses that indicated “no impact, minor impact, moderate impact, major impact and not sure.” Missing responses and responses including “prefer not to say” and “not sure” were excluded from the analysis.

Based on Westbrook et al.’s primary analysis, the characteristics that were protective of staff members from being exposed to or negatively impacted by unprofessional behaviors were, being male and being over 55 years of age. Further analysis undertaken on the data related to the factors that influence speaking up among hospital staff indicated that staff who have worked at the hospital site for over 6 years and who work at private rather than public hospitals may also face lower rates of exposure to unprofessional behaviors (16). The scoring strategy presented in Table 1 is based on well-established bivariate relationships which have been shown in other studies to be associated with higher prevalence of bullying etc. (17–21). Composite scores were used to determine vulnerability profile scores for each respondent, where lower scores indicated a higher degree of protection from exposure to unprofessional behaviors and higher scores indicated higher vulnerability.

**Table 1:** Scoring strategy to determine respondents’ characteristics for (a) vulnerability profile for respondents based on their biographical characteristics, (b) degree of exposure to rudeness, and (c) the negative impact on respondents’ wellbeing because of exposure to rudeness from other hospital staff.

*Vulnerability profile score* (*p*)–Total score for biographical characteristics that increase vulnerability to exposure to unprofessional behaviors, such as age, gender, length of employment at hospital site, age, and funding model for hospital site at which respondents work		
Category	Number of respondents	Score allocated
*Gender (g)*		
Male	299	0
Female	237	1
Prefer not to answer	9	Not included in analysis
Other	1	1
*Age (a)*		
≥55+	105	0
18–54	428	1
Missing	2	Not included in analysis
Prefer not to answer	11	Not included in analysis
*Professional sub-role, if available (s)*		
Career/Hospital Medical Officer/Medical Fellow/Registrar	119	1
Intern/ Resident	98	2
Medical Staff Specialist/Surgical Staff Specialist/Visiting Medical Officer	206	0
Missing	89	Average score from responses provided by 423 respondents calculated as 0.7, and assigned to 89 respondents whose sub- roles are missing

*Hospital funding type* (f) – data from employees who worked across seven hospitals, comprising two public hospitals and five private hospitals		
Public hospital employees	381	1
Private	145	0
*Length of employment at site (l)*		
<1 years to <6 years	283	1
≥ 6 years	258	0
Missing	5	Not included in analysis

*Vulnerability profile score range: maximum possible score of 6 and minimum possible score of 0*		
*Degree of exposure to rudeness* (e) – Respondents who answered the question, *“In the past 12 months, how often have you experienced the following staff behaviors in this hospital – this has happened to me – being spoken to rudely”*		
Never	136	0 (Group 0)
1–2 times / year (assumed average of one instance a year)	140	1 (Group 1)
Every few months (assumed average of one instance a quarter)	96	4 (Group 2)
Around monthly (assumed average of one instance a month)	77	12 (Group 3)
Weekly (assumed average of one instance per week)	65	52 (Group 4)
Daily (assumed average of one instance per weekday at the rate of five working days every week for the whole year)	22	260 (Group 5)
Multiple times daily (assumed average of at least two instances per weekday at the rate of five working days every week for the whole year)	9	520 (Group 6)
Missing	1	Not included in analysis

*Negative impact (i) – Respondents who answered the question, “Thinking about your experience of unprofessional staff behaviors in this hospital, to what extent do you believe they have had a negative impact on: You and your wellbeing”*		
No impact	186	0
Minor impact	199	1
Moderate impact	99	2
Major impact	51	3
Not sure	11	Not included in analysis

Scores calculated for each employee were aggregated and visualized using Microsoft Excel to generate a representation of the distribution of scores and relationships between groups of employees according to vulnerability profile (p), negative impact on wellbeing (i) and extent of exposure to rudeness (e).

## 3. Results

### 3.1. Staff vulnerability to being exposed to incivility

**Figure 1:**
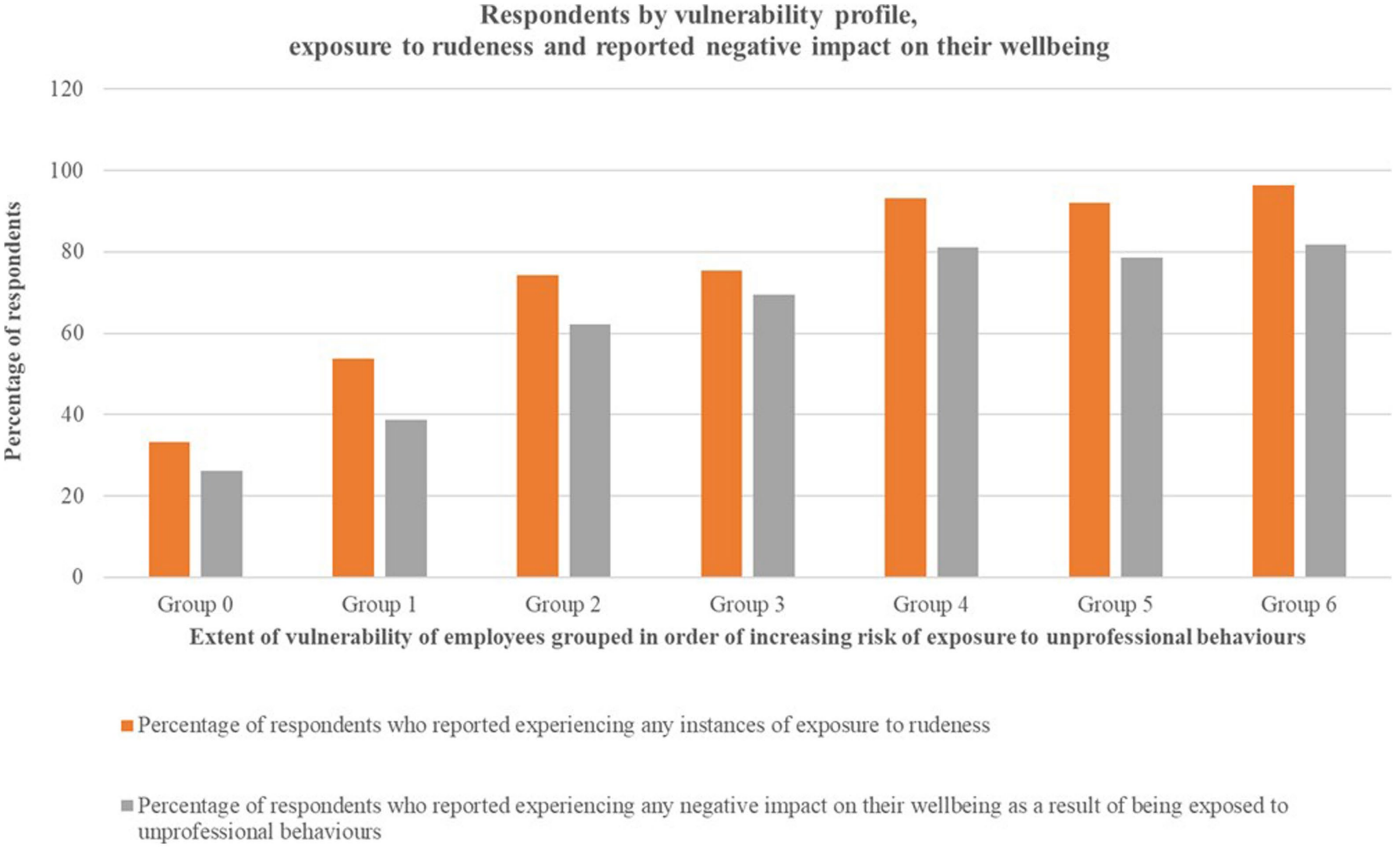
Visualization of proportional increase in percentage of respondents exposed to rudeness against increasing vulnerability profile scores, and percentage of staff who reported any negative impact on their wellbeing because of being exposed to unprofessional behaviors.

Based on the scoring strategy presented above, a total of 512 respondents reported all information required in Table 1 and were included in the analysis. Respondents were scored and grouped by their vulnerability profiles, with 0 indicating a low degree of vulnerability of being exposed to rudeness and 6 indicating the highest degree of vulnerability to exposure. Exposure to rudeness and the impact of unprofessional behaviors experienced were examined for each vulnerability category (Table 2). Increasing vulnerability profile scores appear to be associated with increased exposure to rudeness as well as increased negative impact on wellbeing (Table 2; Figure 1).

**Table 2:** Number of respondents categorized by vulnerability profile scores and corresponding exposure to rudeness and negative impact because of being exposed to unprofessional behaviors.

Vulnerability profile score groups	Percentage of respondents who reported any instances of exposure to rudeness	Percentage of respondents who reported any negative impact on their wellbeing because of experiencing unprofessional behaviors	Total respondents withineach vulnerability profile group
0	14 (33.33%)	11 (26.19%)	42 (8.2%)
1	43 (53.75%)	31 (38.75%)	80 (15.63%)
2	55 (74.32%)	46 (62.16%)	74 (14.45%)
3	74 (75.51%)	68 (69.39%)	98 (19.14%)
4	69 (93.24%)	60 (81.08%)	74 (14.45%)
5	82 (92.13%)	70 (78.65%)	89 (17.38%)
6	53 (96.36%)	45 (81.82%)	55 (10.74%)
Total respondents	390 (76.17%)	331 (64.65%)	512 (100%)

### 3.2. Exposure to incivility and negative impact on staff wellbeing

**Figure 2:**
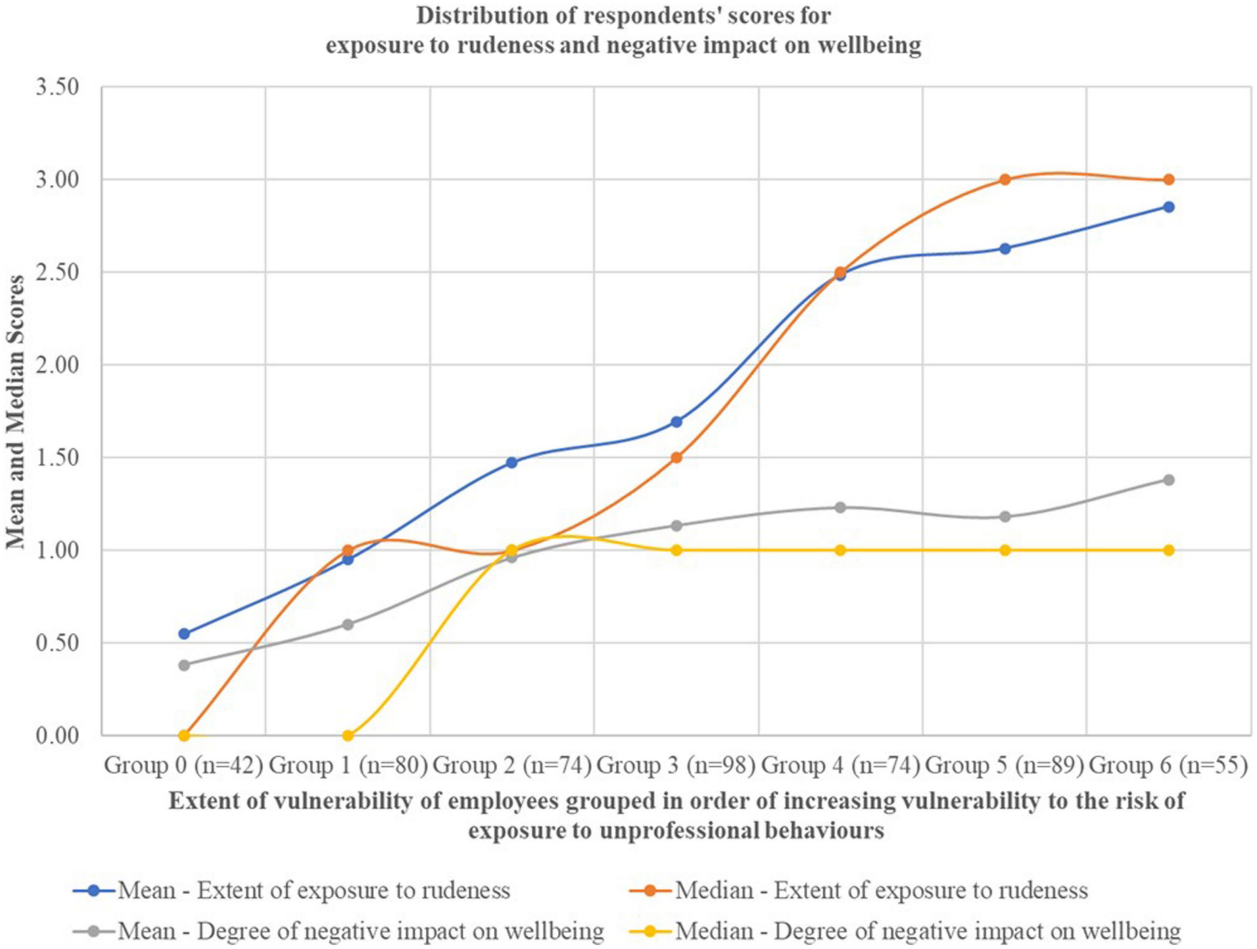
The mean and median scores for extent of exposure of rudeness and degree of negative impact on wellbeing against groups of employees ranked in increasing order of vulnerability profile.

The distribution of scores for 512 respondents were aggregated by exposure to rudeness into seven groups. As described in Table 1, the seven degrees of exposure to rudeness increased incrementally from “never” to multiple times daily.” These groups were labeled Group 0 through to Group 6 indicating increasing frequency of exposure. Scores were aggregated and the means and medians calculated for degree of negative impact on wellbeing and extent of exposure to rudeness and plotted against groups of respondents based on vulnerability profile scores. The pattern of distribution of scores is presented in Table 3 and visualized in Figure 2. Based on the distribution of scores, the relationships between the three categories – staff vulnerability to being exposed to rudeness and frequency of exposure to rudeness appears to increase in proportion to vulnerability score. A negative impact on wellbeing appears to be present for all groups who experienced any instances of rudeness. The only group who appears to report no negative impact on their wellbeing are those who have a combination of protective factors such as age, gender (being male), working at a private hospital and working in a function that affords higher professional status.

**Table 3:** Distribution of scores for degree of negative impact on wellbeing and extent of exposure to rudeness by respondents’ vulnerability profile.

Distribution of scores based on:	Extent of exposure to rudeness in order of increasing exposure (range: 0–6)		Degree of negative impact on wellbeing because of experiencing unprofessional behaviors (range: 0–3)	
Vulnerability profile score that indicates increased risk of exposure to unprofessional behaviors (range: 0–6)	Mean	Median	Mean	Median
Group 0 (*n* = 42)	0.55	0.00	0.38	0.00
Group 1 (*n* = 80)	0.95	1.00	0.60	0.00
Group 2 (*n* = 74)	1.47	1.00	0.96	1.00
Group 3 (*n* = 98)	1.69	1.50	1.13	1.00
Group 4 (*n* = 74)	2.49	2.50	1.23	1.00
Group 5 (*n* = 89)	2.63	3.00	1.18	1.00
Group 6 (*n* = 55)	2.85	3.00	1.38	1.00

Based on the distribution of scores for vulnerability profile, exposure, and negative impact for respondents’ (Table 3; Figure 2), it appears that the vulnerability profile of respondents is indeed proportional to the extent of exposure to rudeness as well as the negative impact on wellbeing reported by respondents. This relationship can be represented as:


p∝[e,i]

Thus, it appears that vulnerability characteristics such as higher age, identifying as male, and working for over 6 years at a private hospital in a role that affords higher influence or professional status, may be protective factors against exposure to uncivil interprofessional behaviors such as rudeness as well as the negative impact on wellbeing resulting from experiencing these behaviors.

## 4. Discussion

Healthcare organizations usually adopt a risk management and mitigation approach to capture data about incidents where negative impacts may have been experienced by staff or patients in hospitals (22). However, the quality and type of data related to contributing factors captured by these organizational reporting systems and interventions can often be poor (23). The potential for reporting systems to better reflect systemic factors and how they interact, influence or may be embedded in individual and situational factors is an emerging area of interest in healthcare organization and safety studies (24–26). Despite growing evidence indicating that incivility may have impacts on a range of organizational, staff and patient outcomes, interprofessional staff incivility may not always be explicitly identified nor addressed as a factor within risk management reporting systems and tools (27, 28). Recent scholarship has highlighted the limitations of top-down culture change interventions within healthcare organizations, owing to the systemic challenges within highly stratified healthcare organizations where multiple subcultures coexist (29, 30). Some structural factors that impact staff behavior may include type of hospital funding, consequent staff and service mix, human resourcing models, and resulting patient care capacity differences between private and public hospitals (31). These elements create different working conditions, dynamics and contextual factors that may influence interprofessional and interpersonal staff behavior (32). Therefore, researchers have argued for the need to synthesize a wider range of theories to improve current healthcare organizational risk management approaches (33). A strength of our study is that it demonstrates how such syntheses may be achieved, by using theory-driven information processing approaches to better understand incident and risk reports that hospitals record about staff unprofessional behaviors. We postulated that medical staff in hospitals are differentially exposed to and impacted by unprofessional behaviors, and that their biographical profiles that are a combination of demographic characteristics as well as contextual factors such as hospital funding type and length of employment, may predispose them to being exposed to negative behaviors. While we have not captured the entire loop of factors that impact interactions between staff when negative incidents unfold, nor all the contextual influencers that contribute to the dynamics observed among hospital staff, our preliminary results demonstrate that artifacts of complexity that present as negative behaviors and their flow-on effects can indeed be captured within an information model. We demonstrated that the experience of being targets of unprofessional behaviors may also point to a pattern of behavioral self-organization for medical professionals. This self-organization appears to coalesce despite recommended organizational and professional codes of conduct and regulatory policies that enshrine positive values-based behaviors within these occupations (34). Our findings also provide empirical evidence to support prior studies that argued that the overarching benefits or disadvantages that stem from pre-existing socio-cultural stratification may overflow into professional interactions on micro, meso, and macro scales (35–38). Therefore, a differential approach to achieving equitable wellbeing outcomes for medical staff and other members within healthcare organizations may be required to counteract the negative and uneven prevalence of unprofessional behaviors in hospitals, as well as the consequent negative impacts because of exposure to these behaviors. Finally, this work can be used as foundational evidence to design differentiated training and development approaches as well as automated monitoring and accountability initiatives to disincentivize unprofessional behaviors that have been normalized within sub-groups of medical professionals. Limitations of this study are that it is exploratory in nature, and a range of potentially relevant factors such as race, class, ethnicity, nationality, residential location, immigration status, self-identified cultural identity and type of employment that may indicate economic precarity (casual, contract work arrangements) have not been captured, and were not built into the study design. Further rigorously designed and more sustained research is warranted to test and validate whether this modeling approach can be used to capture and depict the dynamics of negative behaviors that are part of the cultural features of wider groups of healthcare professionals, organizations, and across diverse geographical and socio-political contexts.

## Data availability statement

The original contributions presented in the study are included in the article/supplementary material, further inquiries can be directed to the corresponding author.

## Ethics statement

The studies involving human participants were reviewed and approved by Human Research Ethics approval was granted by St Vincent’s Hospital Melbourne Human Research Ethics Committee for a multi-site study (HREC/17/SVHM/237). All participants were over the age of 18, employed at one or more of the study sites, and provided informed consent by agreeing to participate in the LION survey. All methods were carried out in accordance with relevant guidelines and regulations. The patients/participants provided their written informed consent to participate in this study.

## Author contributions

JW led the data collection for this article. AP conceptualized and led the analysis reported in this article with input from LL, JW, and RM and drafted the manuscript. All authors reviewed, revised and approved the final version of the manuscript.

## Funding

This research was funded by the National Health and Medical Research Council (Grant ID: 1134459). AP also received funding for this work through the Australian Research Training Program (RTP) scholarship allocation number 2018378/20191504.

## Conflict of interest

The authors declare that the research was conducted in the absence of any commercial or financial relationships that could be construed as a potential conflict of interest.

## Publisher’s note

All claims expressed in this article are solely those of the authors and do not necessarily represent those of their affiliated organizations, or those of the publisher, the editors and the reviewers. Any product that may be evaluated in this article, or claim that may be made by its manufacturer, is not guaranteed or endorsed by the publisher.
